# Effects of Green Tea and Green Tea Incorporated in Nanoparticle Lyotropic Liquid Crystal on Exercise Adaptations: A High-Intensity Interval Training Pre-Clinical Study

**DOI:** 10.3390/nu14153226

**Published:** 2022-08-07

**Authors:** Vitor Nieri, Juliana Ferreira de Souza, Talita Cristina Mena Segato, Érika Leão Ajala Caetano, Fernanda Gomes Leite, Marco Vinícius Chaud, Denise Grotto

**Affiliations:** 1 Laboratory of Toxicologycal Research, University of Sorocaba, UNISO, Sorocaba 18023-000, SP, Brazil; 2 Laboratory of Biomaterials and Nanotechnology, University of Sorocaba, UNISO, Sorocaba 18023-000, SP, Brazil

**Keywords:** sport nutrition, green tea, antioxidants, nanostructured system, physical activity, high-intensity interval training

## Abstract

Green tea (GT) is a natural antioxidant, sensitive to oxidation after preparation. Lyotropic liquid crystals (LLCs) are nanostructured systems used to incorporate bioactive compounds. High-intensity interval training (HIIT) is a workout modality that increases the production of reactive oxygen species (ROS). Thus, this research aimed to compare the effects of GT and GT loaded in LLC in animals subjected to HIIT, considering hematological, biochemical and histological parameters, redox status, and body mass. Monoolein, GT in infusion and Poloxamer 407 were mixed to obtain nanoparticles of LLC (NP-LLC). Healthy male rats were randomized into six groups (*n* = 6/group): Control (C), GT, GT-NP-LLC, Exercise (Ex), GT+Ex, GT-NP-LLC+Ex. Body weight was significantly lower in all groups subjected to HIIT compared to C. The percentages of body mass reduction were 11.3, 13.0, 10.0 and 11.0% for Ex, GT+Ex, GT-NP-LLC and GT-NP-LLC+Ex, respectively, compared to control. GT-NP-LLC and Ex reduced triglycerides compared to C. GT and GT-NP-LLC supplementation combined with HIIT presented higher muscle hypertrophy (25 and 21%, respectively), better physical conditioning, and reduced body weight gain rate compared to HIIT by itself. Moreover, the effects of GT-NP-LLC itself on body mass and biochemical parameters are promising, suggesting NP-LLC could improve the bioavailability of GT.

## 1. Introduction

*Camellia sinensis* is a plant responsible for three varieties of tea: green tea (GT), black tea and oolong tea. They are differentiated by the extraction and manufacturing process of the leaves and stems. Among the teas from *C. sinensis*, GT is the least handled, which preserves its characteristic color and nutrients [1]. GT is rich in polyphenols, and catechins correspond to more than 70% of them; epigallocatechin-3-gallate being the most prevalent catechin GT [2]. It is a potent natural antioxidant with anti-inflammatory, antimicrobial, anticarcinogenic, antihypertensive, neuroprotective, cholesterol-lowering and thermogenic properties [3]. However, polyphenols from GT, when in infusion, are subject to oxidation after preparation [4], which may reduce their biological action.

Drug delivery systems are techniques used to incorporate bioactive compounds. The systems have the ability to deliver an active substance more selectively to a specific site and they can improve the frequency of dosing, decrease variability in systemic drug concentrations, and reduce toxic metabolites [5]. Liposomes, proliposomes, microspheres, nanoparticles, polymers and liquid crystals are, among others, examples of drug delivery techniques [6,7].

Liquid crystal (LC) is a state of matter between the crystalline solid and the isotropic liquid. The LC state is divided into thermotropic, dependent on temperature variation, and lyotropic, dependent on a solvent in addition to temperature [8]. Systems based on nanoparticle lyotropic liquid crystal (NP-LLC) have attracted attention due to their ability to incorporate bioactive components in oil or water [9]. In addition, LC structures are found in animals, plants and bacteria [10], showing biomimetic characteristics that make NP-LLC an important strategy for incorporating bioactive substances. Furthermore, the NP-LLC matrix increases the stability and durability of bioactive compounds [11]. In this way, NP-LLC is able to protect teas from oxidation and enhance the antioxidant effect of phenol present in GT, as demonstrated in a previous publication [12].

High-intensity interval training (HIIT) is a type of exercise characterized by the execution of intense movements for short periods, followed by intervals of low intensity exercise [13]. Physical exercise increases the production of reactive oxygen species (ROS) and there is a concern about their possible harmful effects in the body. In addition, a single session of HIIT training alters the redox state when compared to traditional training [14].

The consumption of antioxidants is related to the neutralization of the harmful reactions induced by ROS. However, it is not clear if exogenous antioxidant consumption can suppress or reduce biological adaptations stimulated by exercise [15,16]. ROS produced in response to the stress from exercise seems to be necessary for physiological and cellular adaptations [17]. They activate pathways, such as peroxisome proliferator-activated receptor-gamma coactivator (PGC)-1 alpha, and mitogen-activated protein kinases (MAPK), which may result in mitochondrial biogenesis and muscle hypertrophy [18]. So, ROS suppression could alter the physical conditioning and the consequent adaptation to the training proposed [19].

Based on this ambivalence, we aimed to do the following: (i) evaluate the body’s natural physiological adaptation to the stress of physical exercise in rats supplemented with antioxidants; and (ii) compare the effects of GT in infusion and, for the first time, GT loaded in NP-LLC, which is a new formulation with the potential to increase the GT action, in animals practicing HIIT. This latter was a great challenge.

## 2. Materials and Methods

### 2.1. GT and NP-LLC Preparation

To prepare the infusion, GT from a commercial brand previously analyzed for antioxidant potential [12] was removed from the sachets and the infusion was prepared in the proportion of 1 g of herb to 75 mL of filtered water, at a temperature of 90 °C, for 5 min [20]. For NP-LLC preparation, the green tea in infusion was freeze-dried, followed by dilution in ultrapure water, before being used to obtain the nanostructured system.

NP-LLC was prepared according to Nieri et al., (2020) [12], with some changes: oil, water and surfactant mixture changed from 10:30:60 m/m to 53.6/35.7/10.7 m/m, respectively. These adjustments were made to standardize the concentration of GT and GT loaded in NP-LLC to be administrated in vivo. Animals from GT groups received 2 mL of GT (via gavage), and animals from NP-LCC groups received 0.35 g of NP-LLC contend 2 mL GT. The emulsion-based precursor system of NP-LLC was prepared with monoolein (Myverol 18–99) as oily phase, GT as aqueous phase (after being freeze-dried and diluted in ultrapure water), and Poloxamer 407 as surfactant. The compounds were mixed under heating (50 °C) in an ultrasound bath at 40 kHz frequency (Unique, USC-3300, Indaiatuba, Brazil) for 5 min.

The formulation was analyzed macroscopically and microscopically. The macroscopic evaluation was performed according to Silva et al., (2017) [21], in which the formulation was observed against a dark background and classified macroscopically as viscous system, with translucent or opaque aspect, or liquid system, with translucent or opaque aspect. Polarized light microscopy was used to assess the absence or presence of anisotropy, lamella formation and morphology of LLC. The analysis was performed under a microscope (Nikon, Eclipse E800, Tokyo, Japan) at polarized mode, with contrast in enlarged mode and high exposure resolution adjustment and the micrographs were captured with a camera (Nikon, DS-r 1, Tokyo, Japan) with the aid of NIS-Elements Software. The emulsion was stored at 4 °C up to fifteen days.

### 2.2. Ethics and Animal Care

This research was approved by the Ethics Committee of the University of Sorocaba (CEUA-Uniso), protocol number 15/2019. A total of 36 male Wistar rats weighing between 160–180 g, with 6 weeks old, were purchased from Anilab Animais de Laboratório Criação e Comércio, Paulínia—SP. All procedures followed the ARRIVE (Animal Research: Reporting of In Vivo Experiments) guide for research involving animals [22]. Animals were housed in ventilated, acclimatized, and shavings-lined cages. The temperature and the light/dark cycle were automatically controlled at 22 ± 2 °C and 12 h, respectively. Animals received food and water *ad libitum*. The experiment started ten days after the animals arrived.

### 2.3. Pre-Clinical Experimental Design

Rats were randomly distributed, using a table of random numbers, in 6 groups (*n* = 6/group): Group I—Control (C); Group II—Green tea (GT); Group III—GT loaded in Nanoparticle Lyotropic Liquid Crystal (NP-LLC); Group IV—Exercise (Ex); Group V—Exercise + Green tea (Ex+GT); Group VI—Exercise + GT loaded in NP-LLC (NP-LLC+Ex).

For in vivo administration, 2 mL of GT and 0.35 g of NP-LLC diluted in 2 mL of filtered water were given by gavage on the same days of exercise. The preparation and dilution were performed to match the concentration of infused GT administered to the animals.

### 2.4. HIIT Training Protocol

Animals from Ex groups were exposed to a week of adaptation to water, with gradual increase in load up to the proposed percentile. After adaptation, animals were subjected to HIIT with low volume training protocol, for 5 weeks (Table 1). An addition of 25% (±3 g) of overload on the total weight of the animal were attached to the animal’s chest through a Lycra pouch. Animals performed 5 to 8 series of 30 s of forced swimming followed by jump to breath, with an interval of 1 min of rest among series. The exercises were performed in a vat of water, at 31 ± 1 °C, with sufficient depth to cover the entire body of the animal in upright position.

To calculate the load up, animals had their body weight measured weekly. Aiming to standardize the training sessions and time, a device was manufactured with 6 tubes 20 cm diameter × 60 cm high, on screened metal support. This support was used to place and remove animals from the water at the same time from the same group. The number of hops of each animal was counted.

At the end of the experiment, all animals were euthanized with intraperitoneal overdose of anesthetic ketamine (100 mg/kg) and muscle relaxant xylazine (6 mg/kg). Blood was collected through the ventricle in two tubes: one with EDTA anticoagulant and another one without anticoagulant. Blood in EDTA tube was used for analysis of oxidative stress and hematological markers; serum (tube without anticoagulant) was used for biochemical analysis. Portions of the liver and the left gastrocnemius were collected for histological analysis.

### 2.5. Hematological and Biochemical Analysis

Red blood cell (RBC), white blood cells (WBC), platelets (PLT), hemoglobin concentration (HGB) and hematocrit percentage (HCT) were analyzed using automated Sysmex XS-1000i equipment (Sysmex Corporation, Kobe, Japan).

Biochemical parameters were performed using commercial kits from Roche Diagnóstica^®^. The parameters analyzed in serum were total cholesterol, high density lipoproteins (HDL), triglycerides, glucose, creatinine, urea, aspartate transaminase (AST), alanine transaminase (ALT), and lactate dehydrogenase (LDH). All analysis followed the manufacturer’s protocol.

### 2.6. Redox State Analysis

For total thiols (represented by reduced glutathione—GSH), 150 µL of the blood was hemolyzed using Triton X-100 10% and precipitated with trichloroacetic acid 30%. The samples were centrifuged, and the supernatants were diluted in 1 M potassium phosphate buffer, pH 7.4. Thiolic groups reacted with 5-5-dithio-bis-2-nitrobenzoic acid (DTNB), forming a yellow complex, which was read in spectrophotometer (MultiSpec-1501, SHIMADZU^®^) at 412 nm [23]. The results were expressed in µmol/mL blood.

Glutathione peroxidase (GSH-Px) activity was determined based on the oxidation of NADPH [24]. Blood was diluted in solution containing reduced glutathione, glutathione reductase, NADPH, sodium azide, reacting with 70 μL of H_2_O_2_. GSH-Px activity was monitored for two minutes at 340 nm. GSH-Px activity is proportional to the consumption of NADPH. The data were expressed in µmol NADPH/min/g Hemoglobin.

Catalase activity was based on the decomposition of H_2_O_2_ by the enzyme, over three minutes, monitored at 240 nm. An aliquot of 20 μL blood was diluted in 50 mM potassium phosphate buffer (pH 7.0) and 70 μL of H_2_O_2_ was added, initiating the reaction. A variation constant (κ), related to Hb, helped in the expression of the activity (κ/g Hemoglobin) [25].

Plasma lipid peroxidation was evaluated by the concentration of thiobarbituric acid reactive substances (TBARS), adapted from Ohkawa et al. [26]. Plasma aliquots (150 µL) were hydrolyzed in alkaline pH (3 M NaOH), at 60 °C for thirty minutes. Afterwards, 6% H_3_PO_4_; and 0.8% thiobarbituric acid (TBA) were added, and the samples were bathed at 80 °C for one hour. Peroxidated lipids, and among them malondialdehyde (MDA), reacted with TBA, forming a pink compound, read at 532 nm. Calibration curve using MDA as standard was constructed to assess the concentration of TBARS.

### 2.7. Histological Analysis

Portions of the liver and the left gastrocnemius were collected. Those samples were kept in 10% buffered formalin for 24 h. After that, samples were washed in water, overnight, and then they were kept in 70% ethanol. After fixation step, the tissues were managed in an automated tissue equipment (PT12—O Patologista^®^). The fragments of the tissues were embedded in histological paraffin. Histological sections of the livers and gastrocnemius were performed using a rotary-manual microtome (O Patologista^®^ MR 2014) with 4 µm thick. Then, the slides were stained with hematoxylin and eosin, and mounted with histological resin [27]. Signs of inflammation, disorder or hypertrophy were observed in the hepatocytes. The gastrocnemius analysis was made through area calculation from a random selection of ten muscles fibers per animal in each group, to measure muscle hypertrophy.

### 2.8. Statistical Analysis

The data were expressed as mean ± standard deviation (SD), and they were tested for homoscedasticity with the Bartlett test. Homogeneous data were analyzed using the One-way ANOVA test for comparison among groups. Tukey or Duncan post-hoc tests were performed to check differences. The statistical analysis of the body weight and the number of jumps of each group over the weeks were performed using two-way ANOVA test. Probability (*p*) values less than 0.05 (*p* < 0.05) were considered statistically significant. All data were performed using Statistica^®^ (version 11.2, College Station, TX, USA) and Graph Pad Prism^®^ software (version 6.0, Graph–Pad, San Diego, CA, USA).

## 3. Results

### 3.1. Body Weight and Physical Conditioning of the Animals

**Figure 1:**
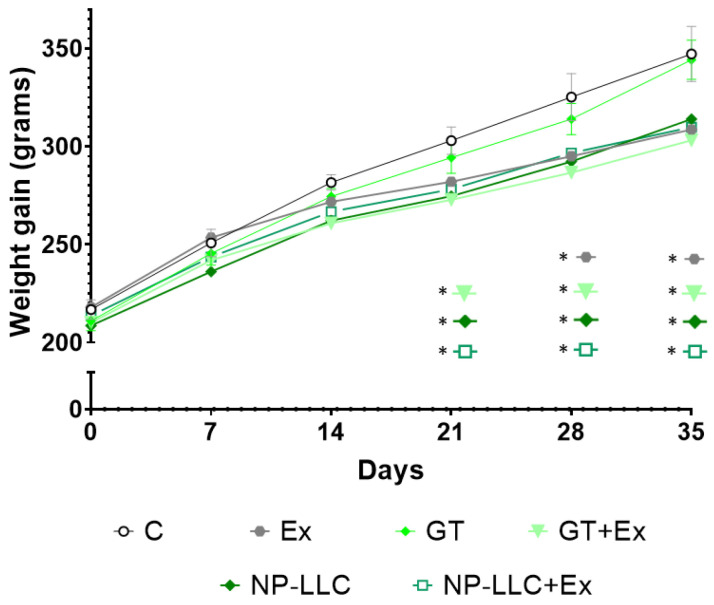
Evolution of the animals’ body mass over the weeks. Note: * *p* < 0.05 when compared to Control.

The body mass of the animals is reported in Figure 1. Initially, animals had similar weight, showing success in randomizing the groups. After the second week of training, there was a decrease in body weight gain in the GT+Ex and NP-LLC groups (not statistically significant, but important, with *p* = 0.062 and 0.075, respectively), compared to C. After three weeks, these trends became significant differences and the NP-LLC+Ex group also showed significant reduction in body weight gain. Comparatively, the weight gain was 11.1% and 9% lower, respectively, in the GT+Ex and NP-LLC+Ex groups compared to control. At the end of the fourth week of intervention, the Ex group showed significant difference compared to C. At the end of the fifth week, the differences pointed out from the third and fourth week remained. GT alone did not present differences compared to control.

Regarding physical training, the number of jumps per animal was counted in all exercise days. The average of jumps within each group, per week, is reported in Table 2. The jump was also used as indicator of physiological adaptations to exercise. Over the weeks, and especially in the last week, there was a significant increase in the number of jumps in all groups under exercise.

### 3.2. Hematological and Biochemical Analysis

Hematological parameters showed no significant differences in the comparison among groups (data number no reported), demonstrating safety for the use of NP-LLC.

**Figure 2:**
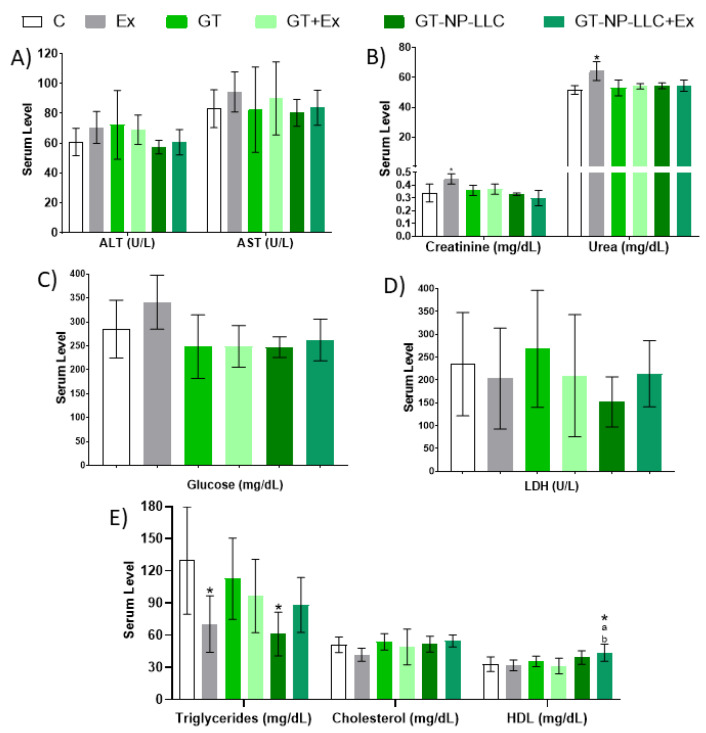
Biochemical evaluation. (**A**) Liver profile; (**B**) Renal profile; (**C**) Glucose concentration; (**D**) Lactate dehydrogenase (LDH) activity and in (**E**) Lipid profile. Note: Data reported by mean ± SD. Liver profile is represented by alanine aminotransferase (ALT) and aspartate aminotransferase (AST); Renal profile is represented by creatinine and urea; Lipid profile is reported by triglyceride, total cholesterol and high-density lipoprotein (HDL). * *p* < 0.05 compared to C, ^a^ *p* < 0.05 compared to Ex, ^b^ *p* < 0.05 compared to GT+Ex.

Biochemical biomarkers are shown in Figure 2. There was a significant increase in creatinine and urea in the Ex group, a decrease in the triglycerides levels in Ex and NP-LLC compared to the C, and an increase in HDL levels in the NP-LLC+Ex group.

Besides showing no significant differences, the NP-LLC+Ex group presented a tendency for decrease in triglycerides levels when compared to C (*p* = 0.07). A tendency towards its reduction was also found in total cholesterol levels in the Ex group, compared to GT (*p* = 0.066) and NP-LLC+Ex (*p* = 0.0554) ones.

### 3.3. Oxidative Stress

**Figure 3:**
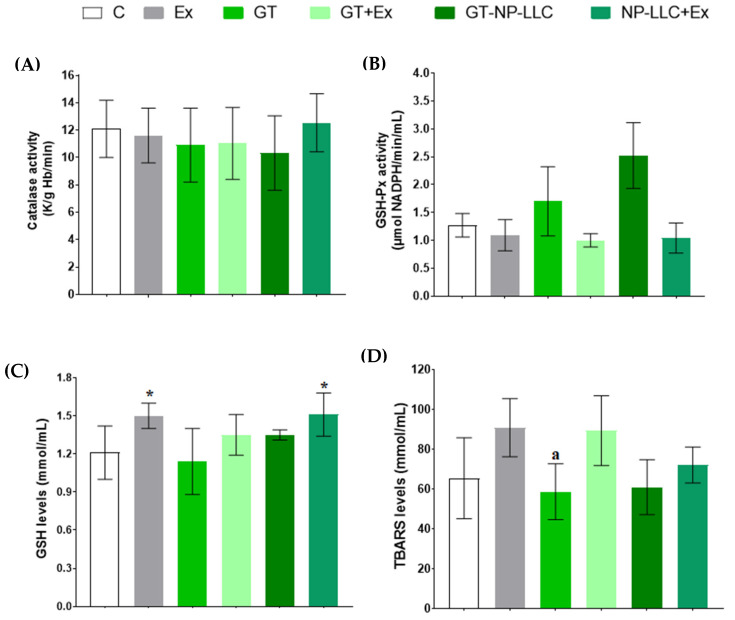
Redox state represented by Catalase (**A**), Glutathione Peroxidase (GSH-Px) (**B**), Reduced Glutathione (GSH) (**C**), Thiobarbituric Acid Reactive Substances (TBARS) (**D**). Note: Mean ± SD * *p* < 0.05 when compared to Control. ^a^ *p* < 0.05 when compared to Ex.

Redox parameters are shown in Figure 3. In addition to the significant differences in the GSH and TBARS biomarkers, there was a trend for increase in TBARS levels in Ex compared to C (*p* = 0.099), and in GT+Ex compared to GT (*p* = 0.052).

### 3.4. Histological Analysis

**Figure 4:**
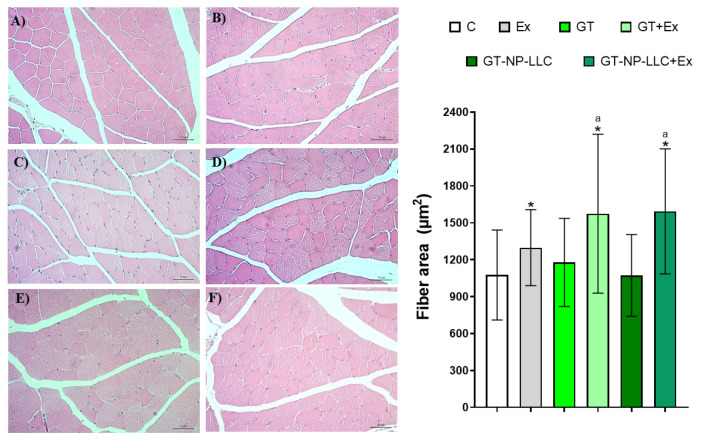
Gastrocnemius histological slices and fiber muscle size of the muscle area: (**A**) Control; (**B**) Green tea (GT); (**C**) GT loaded in nanoparticles of lyotropic liquid crystal (NP-LLC); (**D**) Exercise (Ex); (**E**) GT+Ex; (**F**) GT loaded in NP-LLC+Ex. Note: Mean ± SD * *p* < 0.05 compared to C, GT and NP-LLC, ^a^ *p* < 0.05 compared to Ex.

Fiber size of the gastrocnemius was analyzed to quantify the muscle mass gain of the animals practicing HIIT (Figure 4). All groups practicing exercise showed increase in the diameter of the gastrocnemius muscle fibers compared to C. The Ex-group presented an increase of 22% in the fiber size compared to control, and GT+Ex and NP-LLC+Ex had their fiber size augmented to 53 and 48%, compared to control, respectively. In addition, the GT+Ex and the NP-LLC+Ex groups showed a significant increase in muscle gain (25 and 21%, respectively) compared to Ex group.

**Figure 5:**
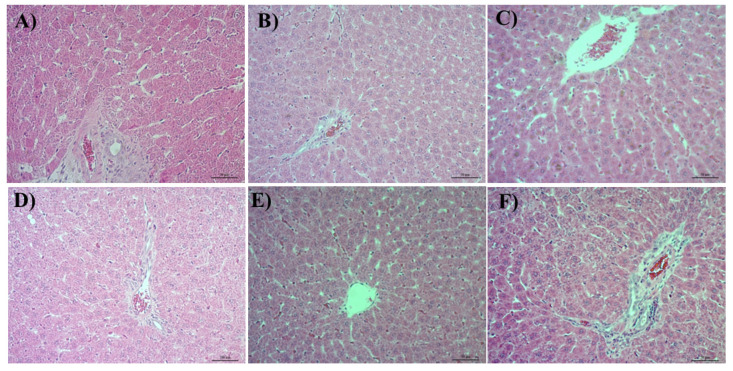
H and E photomicrography (100×) in the region of the hepatic portal system, in which (**A**) Control; (**B**) Green tea (GT); (**C**) GT loaded in nanoparticles of lyotropic liquid crystal (NP-LLC); (**D**) Exercise (Ex); (**E**) GT+Ex; (**F**) GT loaded in NP-LLC+Ex.

Histological analysis of the liver was performed to check liver integrity (Figure 5), especially regarding GT consumption, which has potential hepatotoxic effects when consumed in excess. Since the NP-LLC carrying GT is a new drug delivery system, its impact on the liver is still unknown, and histology can guarantee the non-toxicity assessment of GT loaded in NP-LLC. The findings showed that GT or GT loaded in NP-LLC consumption did not cause hepatocyte disorder or hypertrophy, nor were there any signs of inflammation that could indicate hepatotoxicity.

## 4. Discussion

**Figure 6:**
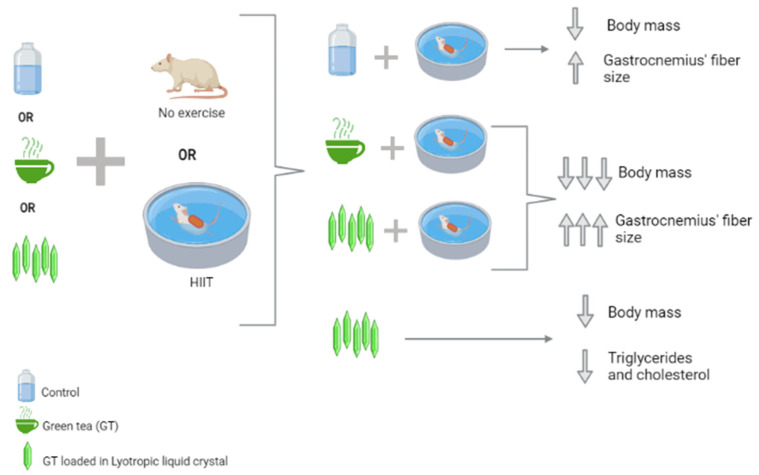
Graphic representation of the main results, summarized in body mass, fiber size and triglycerides and cholesterol levels.

In this pre-clinical study, the administration of GT or GT loaded in NP-LLC anticipated and potentiated the delay in weight gain in groups doing exercise, which might be related to GT consumption having a stimulating effect on metabolism when combined with exercise. A summary of the most important findings is reported in Figure 6.

This result agrees with an 8 week-clinical study, carried out with 30 overweight women, engaged in aerobic predominance training, for three weekly sessions. The clinical study showed that daily consumption of 500 mg of GT extract potentiated the weight loss achieved by exercise, in addition to reducing pro-inflammatory markers and increasing the expression of adiponectin [28]. In another study, 48 overweight men enduring HIIT for 3 weekly sessions, supplemented daily with 750 mg GT extract, presented reduction in body fat after 12 weeks of intervention, compared to control [29].

In both cited studies, GT by itself did not induce weight loss, corroborating our findings. On the other hand, in our study GT loaded in NP-LLC was able to delay body weight gain, as well as reducing triglycerides and increasing HDL levels, without the stimulus of exercise. Since GT loaded in NP-LLC is an original formulation, there are no reports to compare with. However, it is noteworthy that the NP-LLC system is an important technological strategy for incorporating GT, and other compounds, improving stability, and augmenting the bioavailability of active compounds from GT.

Regarding the exercise model, when animals did not stand with their noses above the water surface, they went to the bottom and jumped to the top with their back paws, to breathe. As they improved physical conditioning, they tried to keep swimming. So, we suggest the better conditioned animals kept their heads above the water surface, swimming for longer times, and made less jumps to breathe. In general, there was progress in the physical performance of animals, although this evolution was directly associated with GT or GT loaded in NP-LLC supplementations. There was a progressive increase in the number of series performed over the weeks, so an increase in the number of jumps was expected. However, groups that were supplemented (GT+Ex and NP-LLC+Ex) had slower progressive increase throughout the experiment compared with the Ex group. We hypothesized that the lower the animal’s weight, the higher the muscle hypertrophy observed and the GT stimulating effect on metabolism, improving the GT+Ex and NP-LCC+Ex groups’ physical conditioning.

Concerning the redox course, ROS are known to have an important role in the physiological adaptations stimulated by physical exercise and that antioxidants can scavenge them [16,30]. It is not yet clear whether antioxidant supplementation inhibits or delays the adaptations of physical exercise [31]. In the present study, few differences were observed considering redox parameters. We suggest HIIT was not so stressful for the animals, and they probably had good ROS buffering capacity.

GT consumption potentiated the performance of the animals. In addition, muscle hypertrophy is an important indication of physiological adaptation to training stimulus [32] and an increase in the diameter of the gastrocnemius muscle fibers was observed, being significantly greater in the groups receiving GT or GT loaded in NP-LLC when compared to the Ex group, pointing in the same direction, namely, that the performance of the animals improved with GT and NP-LCC consumption.

Increase in the GSH levels in the Ex group compared to C, combined with lipid peroxidation trend in the same group, showed us the endogenous production of antioxidants in face of exercise stress; this is a natural adaptation of the organism [33]. The increase in GSH might be related to its protective effect and to the reduction of lipoperoxidation, indicating endogenous natural protection of the physiological antioxidant system. A study with CrossFit practitioners supplemented with 500 mg of GT extract reported that after six weeks, the GT supplementation decreased lipid peroxidation and raised total antioxidant capacity compared to placebo [34]. In addition, the CrossFit group showed an increase in plasma uric acid concentration. In our study, the Ex group showed an increase in creatinine and urea rates compared to C. These findings corroborated the higher formation of adenosine monophosphate during the practice of HIIT, which forms uric acid, due to hypoxanthine activation [14].

The reduction in the lipid peroxidation in the GT group compared to Ex, combined with the maintenance of GSH in concentrations similar to C, may be associated with the antioxidant effect from GT in view of the endogenous action of antioxidants. Furthermore, neither GSH nor lipid peroxidation in the supplemented groups were increased, which may suggest the antioxidants present in GT and in GT loaded in NP-LLC were efficient in protecting from lipid peroxidation, without the need to increase endogenous production of antioxidants.

## 5. Conclusions

Green tea is a potent natural antioxidant and its incorporation into the NP-LLC system has been shown to be an important strategy to protect GT active compounds from redox reactions, in addition to increasing its action in vivo. It is not yet clear whether the use of antioxidants is beneficial or disadvantageous to physiological adaptations in physical exercise. Nevertheless, in the present study, supplementation with GT and GT loaded in NP-LLC potentiated the improvement of physical conditioning, increased muscular mass, and reduced the progression of body weight gain of the groups subjected to HIIT. In addition, the effects of GT loaded in NP-LLC on body mass and biochemical parameters are promising and interesting for further investigation in humans.

### Study Limitations

The HIIT protocol did not induce severe ROS production and oxidative stress. Thus, until NP-LLC with GT is a new formulation, it is difficult to properly discuss the results found in this investigation.

## Figures and Tables

**Table 1:** Training evolution over the 5 weeks of the experiment, considering the three exercise groups.

Week	Sessions	Series per Session	Time	Break
1	3	5	30″	1′
2	4	5	30″	1′
3	3	6	30″	1′
4	3	7	30″	1′
5	2	8	30″	1′

**Table 2:** Mean ± SD of the jumps of the animals under training, over the weeks.

Num Jumps	Week 1	Week 2	Week 3	Week 4	Week 5
Ex	18.5 ± 7.7	25.0 ± 8.3	26.2 ± 7.0 *	32.3 ± 14.5 *	43.6 ± 16.0 *
GT+Ex	18.9 ± 16.3	28.1 ± 16.6	29.2 ± 22.6	30.3 ± 18.0	41.2 ± 29.6 *
NP-LLC+Ex	19.2 ± 11.3	26.6 ± 13.7	27.7 ± 11.2	30.5 ± 13.2 *	46.2 ± 27.4 *

Note: * *p* < 0.05 when compared to week 1 of the same group.

## Data Availability

Data and publication materials are available from de corresponding author on a reasonable request.
